# Structural Characterization of EnpA D,L-Endopeptidase from *Enterococcus faecalis* Prophage Provides Insights into Substrate Specificity of M23 Peptidases

**DOI:** 10.3390/ijms22137136

**Published:** 2021-07-01

**Authors:** Piotr Henryk Małecki, Paweł Mitkowski, Elżbieta Jagielska, Karolina Trochimiak, Stéphane Mesnage, Izabela Sabała

**Affiliations:** 1 International Institute of Molecular and Cell Biology, 02-109 Warsaw, Poland; pimalecki@man.poznan.pl (P.H.M.); pmitkowski@iimcb.gov.pl (P.M.); ejagielska@iimcb.gov.pl (E.J.); ktrochimiak@iimcb.gov.pl (K.T.); 2 Department of Molecular Biology and Biotechnology, University of Sheffield, Sheffield S10 2TN, UK; s.mesnage@sheffield.ac.uk

**Keywords:** peptidoglycan hydrolase, M23 peptidase, *Enterococcus faecalis*, endopeptidase

## Abstract

The best-characterized members of the M23 family are glycyl-glycine hydrolases, such as lysostaphin (Lss) from *Staphylococcus simulans* or LytM from *Staphylococcus aureus*. Recently, enzymes with broad specificities were reported, such as EnpA_CD_ from *Enterococcus faecalis*, that cleaves D,L peptide bond between the stem peptide and a cross-bridge. Previously, the activity of EnpA_CD_ was demonstrated only on isolated peptidoglycan fragments. Herein we report conditions in which EnpA_CD_ lyses bacterial cells live with very high efficiency demonstrating great bacteriolytic potential, though limited to a low ionic strength environment. We have solved the structure of the EnpA_CD_ H109A inactive variant and analyzed it in the context of related peptidoglycan hydrolases structures to reveal the bases for the specificity determination. All M23 structures share a very conserved β-sheet core which constitutes the rigid bottom of the substrate-binding groove and active site, while variable loops create the walls of the deep and narrow binding cleft. A detailed analysis of the binding groove architecture, specificity of M23 enzymes and D,L peptidases demonstrates that the substrate groove, which is particularly deep and narrow, is accessible preferably for peptides composed of amino acids with short side chains or subsequent L and D-isomers. As a result, the bottom of the groove is involved in interactions with the main chain of the substrate while the side chains are protruding in one plane towards the groove opening. We concluded that the selectivity of the substrates is based on their conformations allowed only for polyglycine chains and alternating chirality of the amino acids.

## 1. Introduction

**Figure 1:**
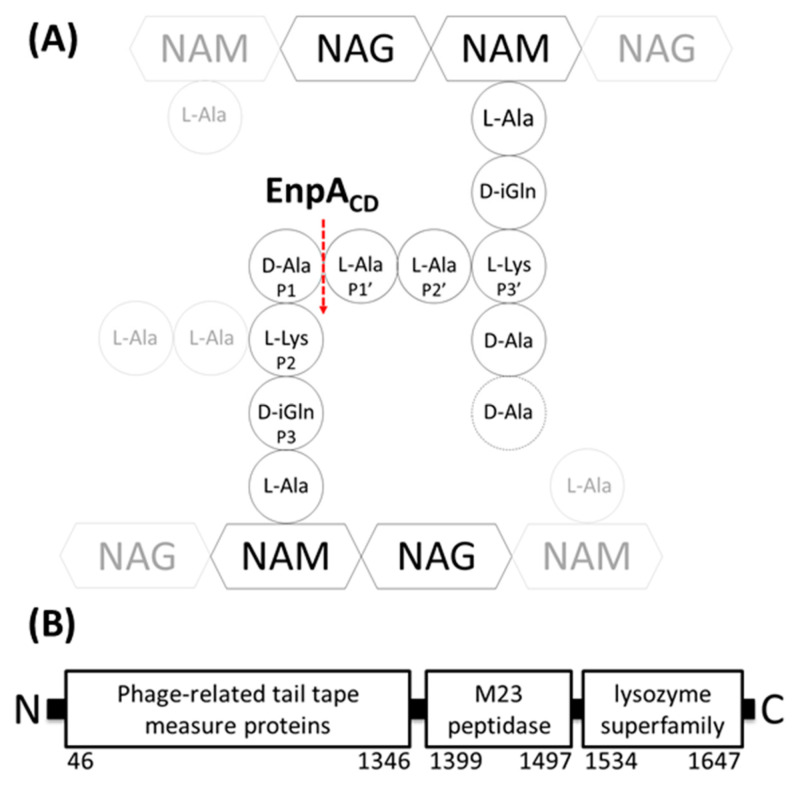
Schematic representations of *E. faecalis* peptidoglycan (**A**) and EnpA modular organization (**B**). (**A**) Cross-linked peptidoglycan monomers (black) and other elements of PG (gray); EnpA_CD_ cleavage site is marked with red dash arrow. (**B**) Domain organization of EnpA; numbers represent amino acid positions in a full-length protein.

Peptidoglycan (PG) is a major component of bacterial cell walls providing cell shape and resistance to the internal turgor pressure. Furthermore, it serves as a scaffold for the attachment of proteins and cell wall anionic polymers, such as teichoic acids. The PG network consists of repeating units of disaccharides, stem peptides and cross-bridges (Figure 1A). Stem peptides are made of both L- and D-amino acids, and their sequence is usually conserved among the genera. They are cross-linked directly in most Gram-negative and in some cases of Gram-positive bacteria such as *Streptococcus oralis* or *Aerococcus viridans*. In Gram-positive bacteria length and composition of the interpeptides vary; e.g., in *S. aureus* they consist of five glycines, while in *E. faecalis* there are two L-alanine residues [1].

Most bacterial genomes encode a large number of hydrolytic enzymes that target virtually every bond present in PG [2]. These enzymes are not only required for the remodeling of normal cell walls during growth and division but also are used as weapons to compete with other bacterial strains living in the same ecological niche. Peptidoglycan hydrolases are used by bacteriophages to enter bacterial cells and to release the progeny at the end of the phage life cycle. Consequently, they are acquiring increasing attention as a potential weapon against pathogenic bacteria, particularly those resistant to antibiotics [3,4,5,6,7,8,9]. Among peptidoglycan hydrolases are endopeptidases belonging to the M23 MEROPS family [10], such as lysostaphin and LytM that cleave pentaglycine cross-bridges present in staphylococcal PG. Members of the M23 family are metallopeptidases containing a zinc ion in their active site, coordinated by two histidine and one aspartic acid residues in the conserved motifs: HXXXD and HXH [11,12,13,14,15]. Some members of this family have also shown to be potent antimicrobial agents that could be applied to eliminate staphylococci, particularly antibiotic-resistant strains [3,4,16,17].

Enzymatic activity of the M23 domain from *E. faecalis* protein called EnpA was reported previously [18]. *E. faecalis* is a Gram-positive bacterium inhabiting the intestinal tract of healthy humans and animals. As an opportunistic pathogen, it can cause various infectious diseases, such as urinary infections, bacteremia and endocarditis [19,20,21]. EnpA is a prophage encoded 1721 residues protein composed of three domains (Figure 1B); the N-terminal domain (1350 amino acids) shares homology with phage tail tape measure proteins. The two domains located towards the C-terminus of EnpA are homologous to domains belonging to PG hydrolases with lytic transglycosylase activity (cleaving glycan chains) and M23 peptidase activity (cleaving PG peptides). The functional analysis of the EnpA catalytic domain (EnpA_CD_) revealed its hydrolytic activity on the D-Ala–L-Ala bond, which is characteristic for *E. faecalis* peptidoglycan, and proposed the minimum substrate of Enp_CD_ to consist of three residues of the donor stem and the amino acids of the cross-bridge [18].

Herein we report a crystal structure of M23 D,L-endopeptidase EnpA_CD_, and discuss its place among other characterized M23 peptidases in terms of the structural–functional relationship. Moreover, we demonstrate the potent lytic activity of EnpA_CD_ against the range of bacterial species.

## 2. Results

### 2.1. EnpA_CD_ Structure Determination

Despite multiple attempts to crystallize and solve the EnpA_CD_ structure, we were unable to obtain crystals of the wild-type protein diffracting below 4 Å; therefore, we generated an inactive variant of EnpA_CD_ H109A. The crystals of this variant gave better diffraction data that were then used to solve the structure of EnpA_CD_ at a resolution of 3 Å.

The crystal structure revealed five protein molecules in the asymmetric unit (ASU). Each single protein molecule covered from 127 to 129 amino acids depending on observed electron density. All EnpA_CD_ molecules in the ASU are similar with root-mean-square deviation (RMSD) values of 0.35 Å–0.65 Å and β-factors for chains A–E ranging from 87 to 108 Å2. A weaker density is observed for the part of the protein described as loop 1 that is partially disordered has the highest B-factor. Thus, further structure description refers to chain A unless stated otherwise.

According to the Matthews analysis, the solvent content of the measured crystal was about 80%. There is only 0.35% of all structures deposited in Protein Data Bank (PDB) that have 80% or more solvent content in the crystal (as of August 2020) [22]. The limited number of intermolecular interactions in high solvent content crystals could be responsible for weak diffraction power and low resolution of the structure herein presented. The crystal packing is presented in Figure S1.

### 2.2. Overall Structure of EnpA_CD_

**Figure 2:**
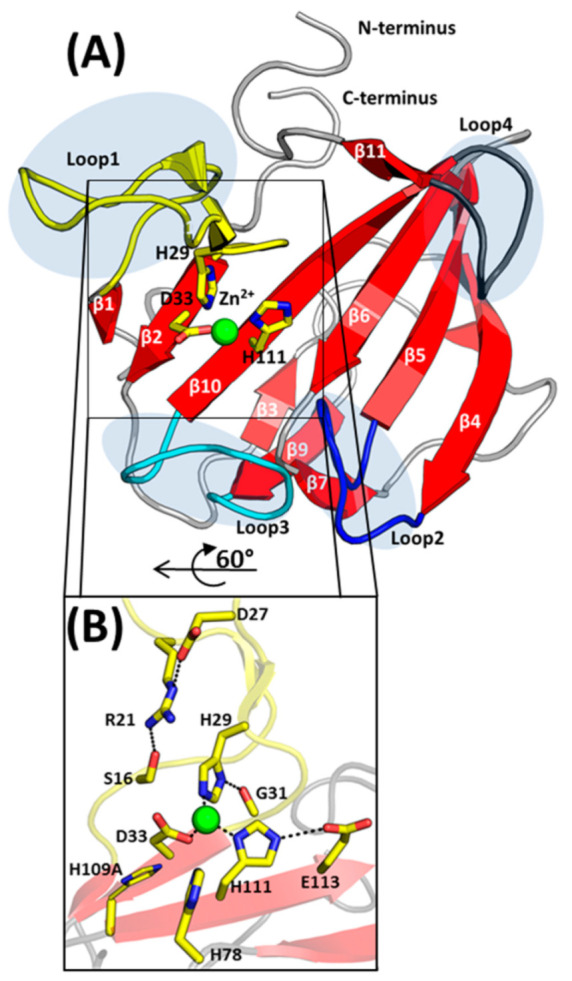
Structure of EnpA_CD_ and its active site architecture. (**A**) Cartoon representation of the secondary structure elements. β-sheets are colored red and labeled; The position of the loops are marked by grey ellipses and colored yellow, blue, cyan and dark grey, respectively, for loops 1–4; other are colored grey. The ligands of the Zn^2+^ ion (green sphere) are shown as yellow sticks and labeled. (**B**) Residues coordinating Zn^2+^ ion and taking part in catalysis are shown as sticks and labeled. Interactions between residues are shown as dashed black lines. The side chain of His109 is marked in gray as it is modeled on a position of Ala residue.

EnpA_CD_ is a globular all β-structure molecule that could be divided into two β-sheets. The smaller 3-stranded β-sheet, composed of strands β3, β9 and β7, runs parallel to the central 7-stranded antiparallel β-sheet. The latter contains a conserved core region for LAS enzymes [15]-strands assigned here as β1, β2, β10 and β6 with zinc chelating residues located on or in close vicinity of β2 and β10 (Figure 2A).

A central β-sheet creates the bottom of the active site groove while its walls are restricted by three out of four protruding loops, as loop 4 rather sticks to the protein core. The loops were described previously in other members of the M23 family: lysostaphin, LytM or LasA [11,12,23] and are analogously assigned herein: loop 1 (Asp17–Gly31, connecting strand β1 and β2), loop 2 (Ser55–Gly61, connecting strands β4 and β5), loop 3 (Gly100–Pro108, connecting strand β9 and β10) and loop 4 (Gly117–Gln122, connecting strands β10 and β11). Loop 1 is the longest among all and participates in interactions with neighboring protein molecules in the crystal. In spite of two short antiparallel β-strands that can be distinguished halfway, the tip of the loop is characterized by the highest B-factor exceeding 150 Å2. The superposition of all molecules in the ASU confirms that loop 1 is the most flexible element in the EnpA_CD_ structure.

### 2.3. The Architecture of Active Site and Binding Groove

The organization of the active site is conserved among the members of the enzymatically active peptidoglycan hydrolases from the M23 family, and a strong electron density corresponding to a metal ion was observed in the close vicinity of His29, Asp33 and His111, residues coordinating Zn^2+^ (Figure 2). The presence of Zn^2+^ ions in the protein was confirmed by an X-ray fluorescence scan (data not shown). The identified zinc ligands are stabilized by interactions with structurally adjacent residues: Nδ of His29 hydrogen binds the carboxyl oxygen of Gly31, while His111 binds Oε of Glu113 (Figure 2B). The insufficient resolution of the data set and high atomic displacement parameters do not allow interpretation of the whole difference electron density close to the Zn^2+^ (Figure S2).

**Figure 3:**
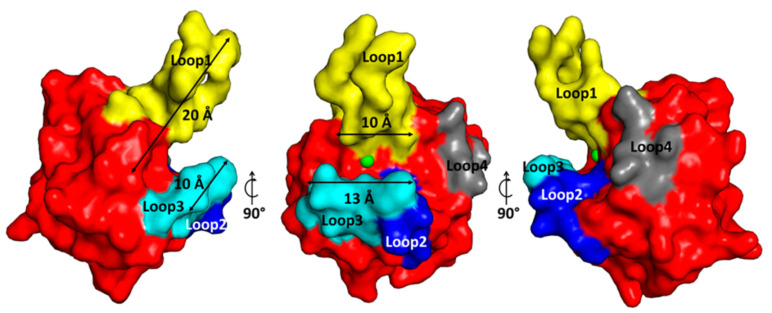
Surface representation of the EnpA_CD_ substrate-binding groove. The colors yellow, blue, cyan and grey depict loops 1–4, respectively. The approximate dimensions of the restricted part of the groove are shown. Loop 4 is positioned close to the protein core rather than taking part in a groove forming. The metal ion is presented as a green sphere.

EnpA_CD_ substrate pocket is placed on the plane of the central β-sheet, and its walls are defined by loops that build a deep (10–20 Å) and very narrow (4–5 Å) groove (Figure 3).

### 2.4. Structural Comparison with Other Members of M23 Family

Chain A of the presented EnpA_CD_ structure was used as a search model in the Dali protein structure comparison server [24]. A structural relationship of Z-score ranging from 10 to 18.4 was found with 15 other protein structures (Table S1). Additionally, an NMR structure of LytU [25] was added as closely related to EnpA_CD_ with RMSD of 1.9 Å. Only entries with defined activity (highlighted with grey background) were used for comparison and further structural analysis.

Two clusters can be distinguished in the superimposed structures; the first is the highly conserved core composed of β-strands that comprise the bottom of the active side (Figure S3a) and a substrate groove which is built of variable loops (Figure S3b). Interestingly, the amino acid sequence conservation in the protein core is restricted only to residues taking part in catalysis and few hydrophobic amino acids.

### 2.5. Specificity and Bacteriolytic Activity of EnpA_CD_

EnpA was shown to cleave bonds between stem peptide’s D-Ala and the cross-bridge’s L-Ala or L-Gly residues [18]. However, no EnpA_CD_ activity was reported on intact PG of bacterial sacculi or whole cells so far.

**Figure 4:**
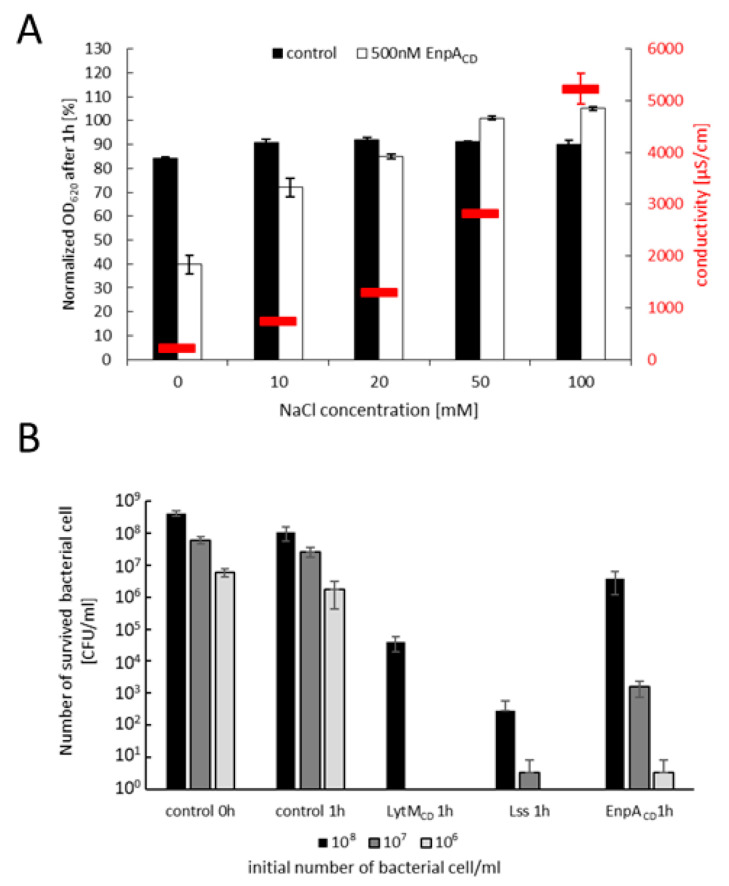
Bacteriolytic activity of EnpA_CD_ on *S. aureus* 8325-4 strain measured in turbidity reduction assay (**A**,**B**) or reduction of CFU/mL (C) (**A**) bacteriolytic activity of 500 nM EnpA_CD_ in buffers of various conductivity (indicated as red lines) (**B**) bactericidal activity of EnpA_CD_, LytM_CD_ and mature lysostaphin presented as number of bacterial cells that survived 1 h incubation with the enzymes (LytM_CD_ 100nM, Lss 100nM, EnpA_CD_ 500 nM) at room temperature starting from 10^6^, 10^7^ or 10^8^ CFU/mL. No enzyme was added to the controls; the number of bacterial cells was determined by plating of serial dilutions on TSB plates. Each experiment was repeated three times using an enzyme from the same preparation and different bacterial cultures. Three technical repetitions were performed each time.

We have defined conditions in which EnpA_CD_ demonstrates a potent bacteriolytic activity on living bacterial cells of diverse species. First, we tested the bacteriolytic activity of EnpA_CD_ in various conditions and concentrations of the enzyme in turbidity reduction assays (Figure 4A and Figure S4). The results demonstrate that EnpA_CD_ has potent bacteriolytic activity in a wide range of temperatures, but only in low ionic strength buffers (Figure 4A,B, Figures S4 and S5). In optimal conditions, 500 nM EnpA_CD_ is able to eliminate up to 10^7^ cells of *S. aureus* at room temperature just in one hour, demonstrating the modest bacteriolytic activity, particularly compared to lysostaphin and LytM_CD_ (Figure 4B).

Once the reaction conditions allowing monitoring of the bacteriolytic activity were established, we verified if the specificity of the enzyme previously determined on short fragments of peptidoglycan [18] would be sustained in assays with whole, live bacterial cells.

We evaluated the bacteriolytic activity of EnpA_CD_ on 32 different bacterial strains of the diverse composition of peptidoglycans and other components of the cell walls (Table S2).

In the cases of direct peptidoglycan cross-linking, such as in *Streptococcus oralis* and *Bacillus subtilis*, no activity was recorded, while low activity was noted against Aerococcus viridans. Moreover, strains with PG cross-linked via D-Asp, *Enterococcus faecium* and *Lactococcus lactis*, were not lysed by EnpA_CD_. A low activity of EnpA_CD_ was observed in the case of *Streptococcus ratti* with L-Thr–L-Ala in the cross-bridge. Moreover, EnpA_CD_ displayed activity against *Micrococcus luteus*. Although classified sometimes as having direct cross-links, *M. luteus* cross-bridge has a composition of the stem peptide: D-Ala-L-Ala-D-Glu(Gly)-γL-Lys-D-Ala-ε-L-Lys [1]. Most of the strains with cross-bridges composed of glycine, serine and alanine residues, either alone or in various combinations, were effectively lysed by EnpA_CD_. Overall, the specificity of EnpA_CD_ demonstrated on isolated substrate fragments is sustained when the whole cells are used in assays conducted in low ionic strength conditions.

Although a strong correlation between the presence of the cross-bridge and EnpA_CD_ activity was recorded, this conclusion is not completely unambiguous. We could observe differences in EnpA_CD_ specificity towards bacterial strains, for which the same composition of cross-bridges is reported indicating that the composition of the cross-bridges is very important but probably not the only determinant of the EnpA_CD_ specificity. EnpA_CD_ cleaves bonds in the junction of donor stem peptide and cross-bridge; thus also stem peptide sequence may play a role in substrate specificity determination.

**Figure 5:**
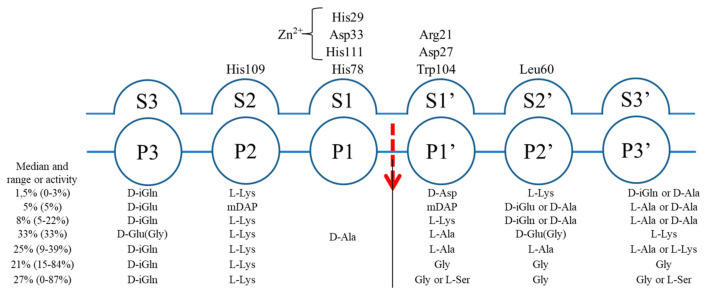
Schematic representation of EnpA_CD_ cleavage site and specificity at particular positions on both sites of the scissile bond (based on the specificity determination presented in Table S2).

According to Schechter nomenclature [26], the positions (P) on the peptide substrate are counted on both sites of the scissile bond (Figure 5). The binding groove is composed of subsites, split into two groups (S and S’) located on both sites of the active center, and each of them accommodates one amino acid of the substrate. D-Ala was present at position P1 of all tested peptidoglycan substrates that were cleaved by EnpA_CD_ [1,18]. Neither our results nor previously published observation on isolated substrates [18] support any straightforward, direct correlation between the presence of particular amino acids at the positions P2 and P3 of the stem and the enzyme specificity.

In the light of the presented results and observations published previously, we can postulate that both, amino acids of the cross-bridge and the stem peptide, might be involved in the determination of the enzyme specificity.

The bacteriolytic activity of EnpA_CD_ can be utilized for various applications. The medical applications of M23 enzymes, such as lysostaphin, are jeopardized by the resistance mechanisms that already exist in nature. One of them is linked to the shortening of the pentaglycine bridge, the other to the substitution of glycines with serines [27,28,29]. We have confirmed that such alteration of the staphylococcal cross-bridge does not hamper the bacteriolytic activity of EnpA_CD_ (Figure S6a,b), and therefore this enzyme can be used in the case lysostaphin resistance spreads.

To check the relevance of other cell wall polymers on EnpA activity, we ran a set of experiments to determine the role of teichoic acids (TA) as an important player in the regulation of peptidoglycan hydrolase activity [30,31]. We observed that the activity of EnpA_CD_ was significantly decreased when bacterial strains were depleted of teichoic acids, e.g., in the *S. aureus* mutant strain lacking TA (ΔtagO) and strains depleted of TA by tunicamycin, an inhibitor of TA synthesis (Figure S6c) [32].

### 2.6. Substrate Binding Model to EnpA_CD_ Reveals Possible Interactions

**Figure 6:**
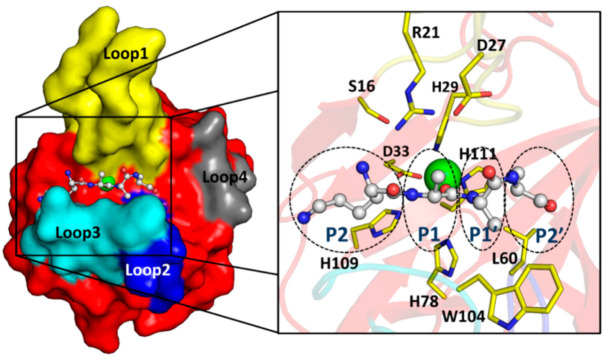
Surface representation of EnpA_CD_ substrate-binding groove with a modeled substrate (L-Lys-D-Ala-L-Ala-L-Ala), shown as stick and ball model. The colors yellow, blue, green and grey shows the loops 1–4, respectively. The right panel shows the zoom of the bonded substrate. Residues speculated to interact with the substrate are shown as yellow sticks. The positions of substrate amino acids are depicted by the dashed ellipses and labeled in dark blue.

Numerous trials to co-crystalize EnpA_CD_ inactive form with various substrate fragments and soaking of preformed crystals with substrates was disappointingly unsuccessful; therefore, we decided to manually dock the substrate (L-Lys-D-Ala-L-Ala-L-Ala) into the binding groove of EnpA_CD_ based on the homology modeling (Figure 6).

P2/S2. The S2 subsite of E. facelis EnpACD, is occupied by the lysine residue of the substrate (P2). The bottom of the S2 subsite is defined by the side chain of His109. There is a branched groove on the S2 subsite that could accommodate lysine residue with a long side-chain; however, the modeled position of the main and side chains might be reversed. It is worth noting that in the case of LytM, the non-primed site of the ligand was also modeled tentatively in two different conformations [33].

P1/S1. The bottom of the S1 subsite is defined by the zinc ion, its ligands and His78. This subsite in EnpA_CD_ is less restricted than in LytM or Lss structures; the Arg21 side chain is moved away from the scissile bond, while in LytM or Lss tyrosine at corresponding position directs its side chain towards the active site. Moreover, EnpA_CD_ S1 subsite widens towards the top and hence is accessible for the side chain of alanine residue, in contrast to Lss where loop 1 might restrict the active site from the top [12,34].

P1′/S1′. The prime subsite, defined by side parts of the loops 1 and 3, becomes broader and rather shallow starting from S1′. In the structure of VanXYg dipeptidase, where Arg74 is present in a position corresponding to Arg21 of EnpA_CD_, the carbonyl oxygen of Ala at P1′ interacts with the Arg74 residue [35]. Similar arrangements of surrounding residues in EnpA_CD_ lead us to propose similar interaction, where carbonyl oxygen of P1 alanine is stretched between Arg21 and Asp27 while its side-chain points towards the hydrophobic ring of Trp104. The S1′ subsite can be extended by the movement of the side chain of Trp104, which is solvent-exposed.

P2′/S2′ EnpA_CD_ has a unique Leu60 residue that contributes to S2′ subsite securing hydrophobic interactions with the 2nd L-Ala side-chain at position P2′ or long aliphatic side-chain of Nε bound L-Lys at P3′. In this part, the binding groove becomes wide and open.

## 3. Discussion

### 3.1. EnpA_CD_ Displays a Potent Antibacterial Activity

Our results demonstrate that EnpA_CD_ has a great bacteriolytic potential that could be used to eliminate certain pathogens, as it was demonstrated for some of the other known peptidoglycan hydrolases, for instance, lysostaphin or LytM [8,36,37,38,39]. The activity of these two aforementioned enzymes is restricted to glycyl–glycine bonds characteristic for staphylococcal cross-bridges. Unlike them, EnpA_CD_ has broader specificity [18], similar to zoocinA (ZooA) from *Streptococcus zooepidemicus* [40].

EnpA_CD_ can effectively lyse living bacterial cells but only in low ionic strength conditions, similarly to the isolated catalytic domain of other members of the M23 family, LytM and lysostaphin [36,41]. Nevertheless, such a limitation can be overcome by attachment of cell wall binding domain, as it was previously demonstrated for LytM catalytic domain fused to CWT from lysostaphin that increased the enzyme affinity to bacterial cell walls, leading to efficient lysis of bacterial cells also in physiological conditions [41,42]. This approach could also be considered to expand the tolerance of EnpA to ionic conditions. Importantly, unlike lysostaphin and LytM_CD_, EnpA_CD_ can accommodate serine at Pl’ or/and P3′positions. 

### 3.2. General Architecture of the Binding Groove of M23 Reflects Enzyme Specificity

**Figure 7:**
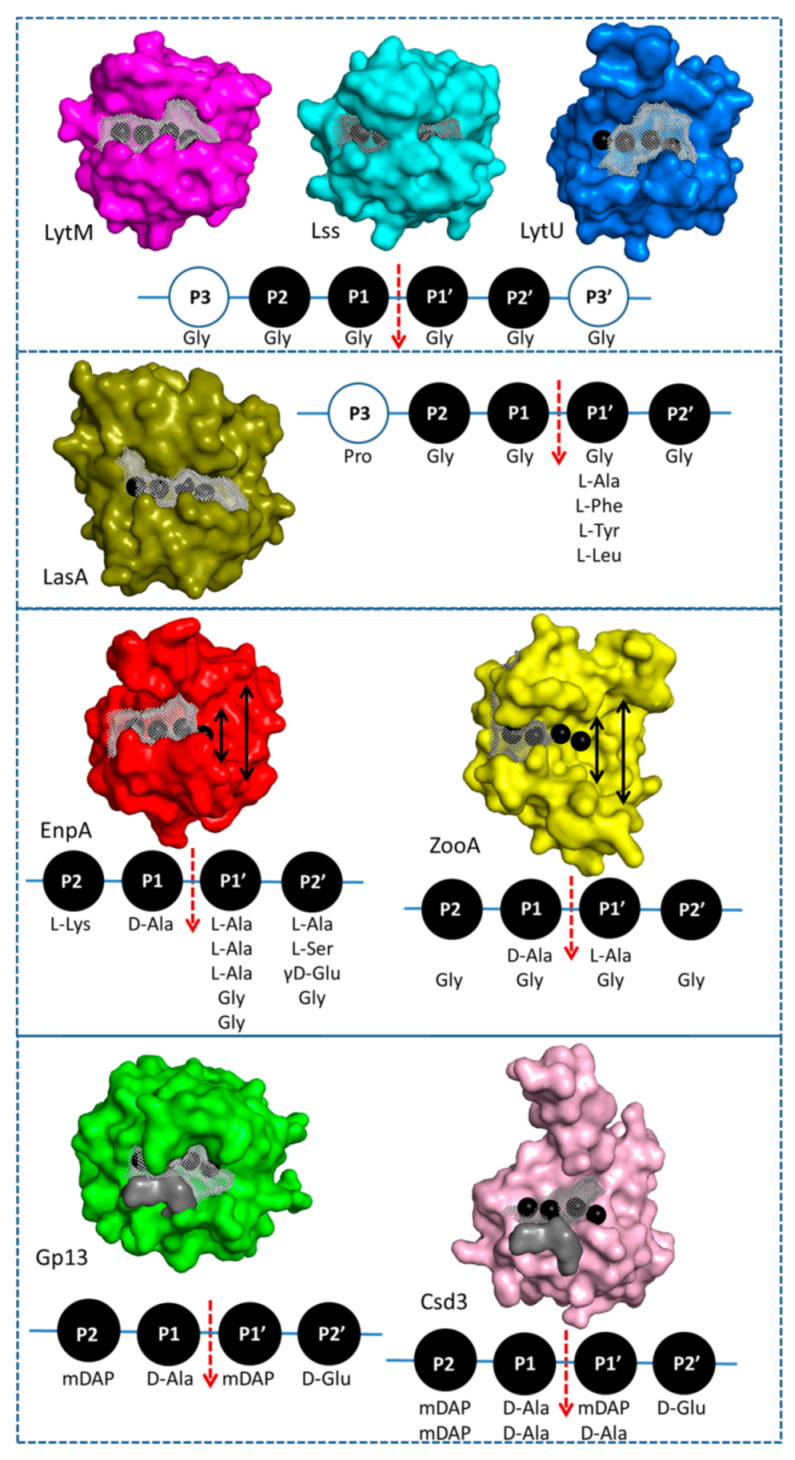
Surface representation of selected proteins from M23 family. The peptide binding sites in the groove are derived from homology modeling of LytM with ligand structure (4ZYB) and are visualized by the black spheres, from left to right from P2, P1, P1′ to P2′. The size and the volume of the binding groove restricted by the neighboring loops are shown by the grey mesh. The volume of the pockets was detected automatically as a potential binding pocket [55] and are 547 Å3, 616 Å3, 472 Å3, 553 Å3, 413 Å3, 404 Å3, 386 Å3 and 275 Å3 for LytM, Lss, LasA, LytU, EnpA, ZooA, Gp13 and Csd,3 accordingly. The grey surfaces in Gp13 and Csd3 supplement the originally unmodelled elements in the structure. They were placed based on the homology modeling and are composed of glycine residues in order to avoid modeling of the side chains. The double-headed arrow in EnpA_CD_ and ZooA structures shows the widening of the substrate-binding groove in the far prime region.

To understand the rules governing the specificity in M23 family enzymes, we analyzed the architecture of binding grooves of newly solved EnpA structure and compared it with structurally related M23 peptidases with reported activity (Figure 7). In M23 proteins, loop 1 and loop 3 build the walls of the active site and create a high and narrow canyon-like entrance on the S1 and S1′ binding subsites with a location of the scissile bond in between. In fact, those subsites look very similar in all the analyzed structures. In contrast, the architecture of the further part of the prime site is much more diverse as a result of the flexibility and substantial structural diversity of loops 2 and 4 [34].

EnpA_CD_ S1 subsite is selective towards D-Ala, the 4th residue of the donor stem peptide (Figure 5). S1′ subsite is less restrictive and can accept such amino acids as glycine, alanine or even lysine via ε amino group but not aspartic acid, while S2′ can accommodate small amino acids (glycine, alanine) or residues with longer side chains but only in D-configuration, often found in bacterial peptidoglycans. The alternating occurrence of L and D amino acids results in an unusual spatial arrangement of the peptides due to increased flexibility of the main chain allowing the side chains to be positioned on one side of the peptide [43]. Only such substrate can fit into a flat binding groove of peptidoglycan hydrolases from the M23 family.

M23 enzymes that share a strong preference towards pentaglycine substrates, such as LytM, lysostaphin and LytU, have elongated, deep and narrow binding grooves [11,12,14,25,33]. The EnpA broader specificity is achieved by opening the binding groove at the prime site making it more accessible for a wider range of substrates [44,45]. Moreover, in contrast to lysostaphin and LytM_CD_, EnpA_CD_ can accommodate serine at Pl’ or/and P3′ positions. This is a unique and important feature as serine at this position results in resistance of the host against lysostaphin and other glycyl–glycine M23 endopeptidases and is a naturally occurring mechanism of resistance to these enzymes [27,28,46,47,48].

The LasA substrate groove is significantly shorter and shallow at the prime subsite what corresponds well to the specificity of LasA that is broaden compared to the aforementioned enzymes [23]. The LasA enzyme was shown to tolerate aromatic or branched amino acids at the P1′ position, however not at P2′ [23,49]. The substrate grooves of Gp13 and Csd3 enzymes are less restricted at the prime subsite. Both enzymes cleave peptide bond between mDAP and D-Ala, and the architecture of their binding grooves shows that there is enough space at S1 and S2 subsites to accommodate bulkier residue, such as mDAP [50,51,52].

We also analyzed the binding of similar substrates by structurally related enzymes from the LAS family [15]. Substrate binding studies of the VanXY protein have shown that the full tetrapeptide of a donor stem peptide is needed for optimal activity [35], similarly to previously reported data for minimal EnpA_CD_ substrate [18]. The data show that in both, EnpA_CD_ and VanXY, the stem peptide interacts with the part of protein close to S2 binding subsite and that there is great variability of substrates residues in P1′ position. VanX displays activity on D,D- and D,L- dipeptides (D-Ala-D-Ala; D-Ala-D-Phe; D-Ala-L-Gly, DL-Ala-DL-Ser, DL-Ala-DL-Val; DL-Ala-DL-Asn) but is not active against tripeptides and L,L-dipeptides [53].

Some similarities in binding groove architecture are also observed in other enzymes with similar specificity to EnpA. CHAPK domain of the endolysin LysK from *S. aureus* bacteriophage K is not a member of the M23 family, but like EnpA, it cleaves bonds between D-Ala of the peptidoglycan stem peptide and a glycine of the cross-bridge. Although the overall structure of this peptidase (pdb ID: 4CSH) is very different, the architecture of the binding groove resembles the one of EnpA [54] (Figure S7).

This analysis brought us to the conclusion that the substrate specificity of the M23 enzymes is determined by the general architecture of the binding groove, which in the proximity of the active site is narrow and deep while its bottom is based on a flat β-sheet. Only interactions between the substrate main chain and the bottom of the groove are possible as the structure does not provide space for the side chains. As a consequence, substrates have to be built either of residues that have no or short side chains and/or have alternating L and D amino acids that increase the main chain flexibility. In other words, the substrate selectivity seems to be based on spatial configurations that allowed for glycine residues and the chirality of amino acids, but the complex structure with the substrate would be needed to confirm these conclusions.

### 3.3. Arginine 21 Plays a Role in EnpA Activity

Apart from the conserved zinc-binding residues, other amino acids were proposed to play a crucial role in M23 enzymes, such as Tyr204 in LytM [33] or Tyr151 in LasA [23]. In some M23 enzymes, the corresponding position is occupied by other residues, such as glutamine in Gp13 or arginine in Csd3 and EnpA_CD_ (Figure S8). Arginine residues are also found at this position in other enzymes grouped together with M23 as the LAS family [15], which also comprises enzymes cleaving D-Ala–D-Ala bonds (carboxypeptidase from *Streptomyces albus*, PDB id: 1LBU [56], peptidase VanY from *E. faecium*, PDB id: 5HNM, VanXYg dipeptidase from *E. faecalis*, PDB id: 4MUQ [35] or VanXYc from *Enterococcus gallinarium* (PDB id: 4OAK)).

**Figure 8:**
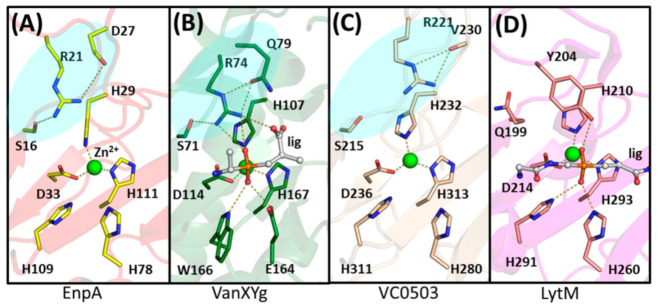
The active site of (**A**) EnpA, (**B**) VanXYg with phosphinate dipeptide (PDB id: 4MUQ), (**C**) *Vibrio cholerae* M23 (PDB id: 2GU1) and (**D**) LytM with phosphinate tetraglycine (PDB id: 4ZYB). There is a striking similarity between residues arrangement around the active site of A, B and C (shaded area). The hydrogen bonds between residues and ligands are shown as dashed lines. Zinc ion is presented as a green sphere.

In the EnpA_CD_ structure, the Arg21 side chain is in close contact with Ser16 and Asp27, suggesting the formation of the hydrogen bond and a salt bridge. The equivalents of these two residues are usually present together also in other M23 proteins, thus might be structurally important as demonstrated for VanXYg, where the amide nitrogen of Gln79, a residue that corresponds to the Asp27 in EnpA, was suggested to be necessary for catalysis (Figure 8) [35]. In LytM, a Tyr204 was proposed to play a role in stabilization of the substrate transition state during cleavage reaction [33], while the guanidinium group of arginine occupying an equivalent position in some carboxypeptidases was proposed to act as electrophilic catalyst interacting with transition state oxyanion [57]. Contrary to the tyrosine residues, arginine can form separate hydrogen bonds, positioning other residues around the active site and stabilizing its geometry while binding the substrate at the same time [57]. Such additional bonds created by arginine are important for EnpA_CD_ activity, as substitution of R21Y inactivated the enzyme but did not influence its structure (Figure S9a,b).

## 4. Materials and Methods

### 4.1. Gene Cloning

The DNA encoding the EnpA_CD_ H109A was obtained by gene synthesis and cloned as a NcoI-BamHI fragment in pET2818.

### 4.2. Mutagenesis

Two single mutations were introduced into the EnpA_CD_ gene: A109H substitution restored the wild type of the enzyme and R21Y generated in the WT background. PCR-based site-directed mutagenesis was performed using Phusion high-fidelity DNA polymerase (Thermo Scientific, Waltham, MA, USA) according to the manufacturer’s 3-step protocol. The plasmids were used to transform *E. coli* TOP10 host cells, and the selected clones were verified by DNA sequencing.

### 4.3. Protein Purification

The expression of wild-type EnpA_CD_ and proteins with H109A or R21Y substitutions was carried out in *E. coli* BL21(DE3). Transformed bacteria were grown in auto-inducting LB broth with 50 µg/mL ampicillin overnight at 25 °C and vigorous shaking. The cells were centrifuged at 4000 rcf for 10 min and then resuspended in buffer A (50 mM TrisHCl pH 8,0, 500 mM NaCl, 20 mM imidazole, 10% glycerol). Bacteria suspension was sonicated on ice for 5 min (cycle: 15 s work, 60 s rest) and centrifuged 145k rcf for 40 min at 4 °C. The supernatant was loaded on HisTrap HP 5ml column (GE Healthcare), washed in buffer A, and gradually eluted to buffer B (20 mM TrisHCl pH 8,0; 200 mM NaCl, 500 mM imidazole; 10% glycerol). Fractions containing overexpressed protein were concentrated and loaded on the 26/600 Superdex 75 prep grade, size-exclusion chromatography column (GE Healthcare). The separation was performed in buffer B without imidazole. The peak fractions were analyzed by SDS-PAGE. Pure protein was concentrated, flash frozen and stored at −80 °C.

### 4.4. EnpA_CD_ H109A Crystallization

EnpA_CD_ H109A crystals were grown using the sitting-drop vapor-diffusion method at 291 K. A crystallization robot (Phoenix, Art Robbins Instruments) was used to mix 0.2 µL of JCSG-plus screen buffers (Molecular Dimensions) with an equal volume of 15 mg/mL protein solution in 50 mM Tris-HCl buffer pH 7.5 and 200 mM NaCl on 96-well crystallization plates (Molecular Dimensions). The crystals appeared after 3 days in a buffer consisting of 0.8 M succinic acid, pH 7.0. Crystallization in this condition was repeated on a 24-well Cryschem Plate (Hampton Research) by mixing 4.0 µL of 9.8 g/L protein solution with 2.0 µL of well solution. Again, the crystals appeared after 3 days and were harvested on 10th day, when they reached the largest size (co-crystallization with pentaglycine was also attempted, but no crystals were obtained). The crystals were cryo-protected by quickly immersing in the well solution mixed in a 1:1 volume ratio with 50% (*v*/*v*) glycerol and flash cooled in liquid nitrogen.

### 4.5. Data Collection, Processing

EnpA_CD_ X-ray diffraction data set were collected at beamline P13 operated by EMBL Hamburg unit at the PETRA III storage ring (DESY, Hamburg, Germany) using the PILATUS 6M detector [58]. The data were integrated and scaled using the XDSAPP software [59]. All relevant data collection and processing statistics are presented in Table S3.

### 4.6. Structure Determination and Refinement

Firstly, the search model of NMB0315 from *Neisseria meningitidis* (PDB ID: 3SLU) [60] was chosen based on the highest amino acid sequence homology. Upon sequence alignment analysis, the model was truncated to residues 294–387 and was used for molecular replacement. According to the Matthews correlation coefficient, initially, 10 protein molecules were searched in the asymmetric unit (ASU), which corresponds to the 57.8% solvent content [61]. However, only two protein molecules were found using the Phaser software [62], indicating more than 91% solvent content in the crystal. A subsequent structure refinement gave high R values: 0.56/0.58 (R_work_/R_free_). In the following phasing round, the protein model predicted by I-Tasser with a primary sequence consistent with the EnpA_CD_ protein [57] was used with similar results. Although the initial R factors were suggesting a completely random solution, the electron density was convincing for both protein molecules and sufficient enough for moderate manual structure refinement using Coot software [63]. The electron density peaks greater than 13σ were identified. Their position close to the active site residues coordinating the Zn ion in a reference structure indicated that the obtained results are at least partially correct. The refined structure was used for the localization of further copies of EnpA_CD_ in the ASU with no success. Therefore, the symmetry was reduced from P6522 to P65, and molecular replacement was performed again. 10 protein molecules were found in the ASU, and initial refinement resulted in R values: 0.37/0.42 (R_work_/R_free_). Further inspection with the Zanuda software indicated that the P65 space group was incorrect and suggested changing it back to P6522. Subsequently, the structure was successfully refined in the higher symmetry with five molecules in the asymmetric unit initially with REFMAC [64] and then with Phenix.refine [65]. The final model was obtained after cycles of manual adjustments using Coot software [63], then validated and deposited in the Protein Data Bank [22] with the accession ID 6SMK. All model refinement statistics are presented in Table S3.

### 4.7. Modelling of the EnpA_CD_ Substrate Complex

To obtain information on the interactions of the substrate with the particular subsites of the enzyme, we docked tetrapeptide substrate consisting of the residues from the stem peptide and the cross-bridge of the *E. faecalis* (L-Lys-D-Ala-L-Ala-L-Ala) in EnpA_CD_ coordinates. The substrate was chosen based on the data about EnpA_CD_ specificity [18]. The substrate was manually docked into the EnpA binding groove based on structural data of phosphinate tetrapeptide bound to LytM [33] and phosphinate dipeptide bound to the VanXYg protein in a manner to place the D-Ala–L-Ala scissile bond in the active site center. 

### 4.8. Enzymatic Activity Assays

Turbidity reduction assays. Bacterial strains were grown in a liquid tryptic soy broth (TSB) medium and collected in a logarithmic phase when the cultures reached OD_620_ of 0.6–0.8. Bacterial cells were centrifuged and suspended in buffer C (50 mM glycine, pH 8.0) to OD_620_ of 2.0. On a 96 well plate, 100 µL of cell suspension was mixed with 100 µL of enzyme in buffer C supplemented with NaCl, whenever needed. The standard concentration of EnpA_CD_ in the reaction mixture was 500 nM unless otherwise stated. In the control samples, bacterial suspensions were mixed with 100 µL of buffer C. OD_620_ of the reaction was monitored in The Infinite^®^ F50 plate reader (TECAN) at room temperature every 10 min for 1 h. Each experiment was repeated three times using an enzyme from the same preparation and different bacterial cultures. Three technical repetitions were prepared each time. The decrease in OD_620_ after one hour was calculated as a percentage of the OD_620_ in the control without enzyme. The difference in the OD_620_ in the control between time 0 and after 1 h incubation is due to autolysis.

CFU/mL reduction assays. Exponentially growing bacteria (OD_620_ = 0.6–0.8) were suspended in buffer C at a concentration between 106–108 CFU/mL depending on the experiments before the addition of the enzyme. At defined time points, 100 µL aliquots were diluted and plated on TSB-agar plates. The number of colonies was counted after overnight incubation at 37 °C, and the CFU/mL calculated. The control reaction was run in parallel but without the enzyme.

### 4.9. FTIR

Tested proteins were purified according to the protocol described above. After the final purification step, enzymes were concentrated on Amicon Ultra filters (10k MWCO). Protein concentrations were 9.5 and 14.3 g/L for wild type and protein with R21Y substitution, respectively. The FT-IR Spectrometer (Bruker, TENSOR 27) was calibrated against filtrate obtained during sample preparation, which allowed to separate the noise (buffer components) from the signal generated by the proteins. For the measurement, 20–30 µL of each protein solution was used. The results were analyzed using OPUS software.

## 5. Conclusions

We defined conditions for effective lysis of pathogenic bacteria by EnpA_CD_, demonstrating a great bacteriolytic potential of the enzyme, which has much broader specificity as compared to the previously characterized counterparts, but its activity is limited by ionic conditions. At the same time, a comparative analysis of the structural and biochemical data of EnpA_CD_ allowed us to describe the structure–function relationship in M23 enzymes. This new information might help in the structure-based engineering of EnpA_CD_ and related bacteriolytic enzymes in order to modulate their specificity and generate desired activity that could be used to eradicate pathogenic bacteria and those resistant to antibiotics or other bacteriolytic proteins.

## Supplementary Materials

The following are available online at https://www.mdpi.com/article/10.3390/ijms22137136/s1: Tables S1–S3, Figures S1–S9.
