# Supplementary material for: Structural Characterization of EnpA D,L-Endopeptidase from Enterococcus faecalis Prophage Provides Insights into Substrate Specificity of M23 Peptidases
Source: Int J Mol Sci. 2021 Jul 1;22(13):7136. doi: 10.3390/ijms22137136 (PMC8269130; doi:10.3390/ijms22137136)

## Supplementary materials

**Table S1.** The match list of structurally similar proteins searched by $EnpA_{CD}$ structure query in Dali server (1-15) and manual search (16). Grey shading indicates catalytic domains of proteins for which the specific activity was demonstrated.

| No: | PDB id | Dali Z score/ RMSD/%id | Protein name and origin | Hydrolytic activity |
|---|---|---|---|---|
| 1 | 2GU1 | 18.4/ 2.3/ 29 | Lysostaphin like peptidase (VC0503) from *Vibrio cholerea* | No data |
| 2 | 3SLU | 17.6/ 1.9/ 39 | Lysostaphin like, outer membrane protein (NMB0315) from *Neisseria meningitidis* | No data |
| 3 | 5J1K | 16.5/ 2.3/ 28 | Cell shape determinant (Csd2) from *Helicobacter pylori* | Not active - degenerated active site |
| 4 | 1QWY | 16.3/ 2.2/ 36 | LytM glycyl-glycine hydrolase from *Staphylococcus aureus* | Active |
| 5 | 4LXC | 16/ 2.3/ 30 | Lysostaphin glycyl-glicine hydrolase from *Staphylococcus simulans* | Active |
| 6 | 2HSI | 16/ 2.1/ 31 | Putative M23 from *Pseudomonas aeruginosa* | No data |
| 7 | 4RNY | 15.9/ 2.6/ 34 | Cell shape determinant (Csd3) from *Helicobacter pylori* | Active |
| 8 | 5J1M | 15.5/ 2.2/ 32 | Cell shape determinant (Csd1) from *Helicobacter pylori* | No data |
| 9 | 3TUF | 15.3/ 2.4/ 29 | Stage III sporulation protein from *Bacillus subtilis* | Not active - degenerated active site |
| 10 | 3NYY | 13.5/ 2.3/ 26 | Putative M23 from *Ruminococcus gnavus* | No data |
| 11 | 5GT1 | 12.6/ 2.2/ 21 | Choline binding protein (CbpA) from *Lactobacillus salivarius str. Ren* | No data |
| 12 | 3CSQ | 11.8/ 2.8/ 16 | Gp13 protein – lysozyme-like from bacteriophage φ29 | Active |
| 13 | 5KVP | 11.5/ 2.7/ 32 | ZoocinA endopeptidase from *Streptococcus equi subsp. Zooepidemicus* | Active |
| 14 | 3IT5 | 10.8/ 2.7/ 22 | LasA protease from *Pseudomonas aeruginosa* | Active |
| 15 | 5b0h-A | 10.8/ 2.8/ 17 | Leukocyte cell-derived chemotaxin2 from *Homo sapiens* | Not active - degenerated active site |
| 16 | 5kqb | 15.4/1.9/33 | LytU peptidoglycan endopeptidase from *Staphylococcus aureus* | Active |

**Table S2.** Specificity of $EnpA_{CD}$. Bacteriolytic activity of $EnpA_{CD}$ was measured as $OD_{620}$ decrease of bacterial suspension collected at logarithmic growth phase, suspended to $OD_{620}$ of 1.0 in 50 mM glycine buffer pH 8.0 with or without 100mM NaCl and treated with 500nM $EnpA_{CD}$ for 1 hour. The results are presented as % of reduction of initial $OD_{620}$ after 1 hour normalized to the control (without enzyme). All assays were run in triplicates and repeated at least twice.

| Bacteria | Source | Cross-bridge connected to D-Ala- | Activity (%) ± standard deviation | |
|---|---|---|---|---|
| | | | ionic strength | |
| | | | low | high |
| *Lactococcus lactis* | CCM 1877 | D-Asp | 0±0 | 6±3 |
| *Enterococcus faecium* | CCM 7167 | | 3±3 | 7±4 |
| *Micrococcus luteus* | ATCC 10240 | L-Ala-D-Glu(Gly)-L-Lys-D-Ala | 32±4 | 0±0 |
| *Bacillus subtilis* | IBB 886 | Direct | 5±2 | 3±3 |
| *Streptococcus oralis* | CCM 7412 | | 7±3 | 2±1 |
| *Aerococcus viridans* | CCM 1914 | | 22±3 | 11±2 |
| *Streptococcus equi subsp. zooepidemicus* | CCM 7316 | L-Ala2-3 | 0±4 | 0±4 |
| *Streptococcus ratti* | CCM 7443 | | 9±8 | 7±6 |
| *Streptococcus pyogenes* | CCM 7418 | | 21±1 | 3±0 |
| *Streptococcus agalactiae* | IBB 130 | | 28±4 | 5±7 |
| *Enterococcus faecalis* | IBB 421 | | 34±10 | 2±4 |
| *Enterococcus faecalis* | CCM 4224 | | 38±9 | 3±3 |
| *Enterococcus faecalis* | CCM 1875 | | 39±10 | 0±0 |
| *Salinicoccus roseus* | CCM 168 | Gly5-6 | 84±2 | 7±9 |
| *Staphylococcus cohnii* | DSM 20260 | | 15±4 | 0±1 |
| *Staphylococcus xylosus* | PCM 2114 | | 16±0 | 0±2 |
| *Staphylococcus lugdunensis* | PCM 2430 | | 40±2 | 1±1 |
| *Staphylococcus agnetis* | DSMZ 23656 | | 19±3 | 0±3 |
| *Staphylococcus carnosus subsp. carnosus* | DSM 20501 | | 20±0 | 0±2 |
| *Staphylococcus aureus* 8325-4 | NCTC 8325 | | 43±6 | 1±4 |
| *Staphylococcus saprophyticus* | ATCC 15305 | (L-Ser-Gly)1-2-Gly3-4 | 78±8 | n.d. |
| *Staphylococcus capitis* | PCM 2121 | | 14±2 | 0±2 |

| | | | | |
|---|---|---|---|---|
| *Staphylococcus pasteuri* | PCM 2445 | | 15±1 | 0±4 |
| *Staphylococcus haemolyticus* | PCM 2113 | | 21±3 | 0±4 |
| *Staphylococcus intermedius* | DSM 20373 | | 22±7% | 2±0% |
| *Staphylococcus warneri* | PCM 2107 | | 29±4% | 0±6% |
| *Staphylococcus simulans* | DSM 20037 | | 87±4% | 0±0% |
| *Staphylococcus simulans* | CCM 3583 | | 65±4% | 4±0% |
| *Staphylococcus epidermidis* | ATCC12228 | | 4±1% | 0±0% |
| *Staphylococcus pettenkoferi* | VCU 012 | | 0±3% | 0±0% |
| *Staphylococcus pettenkoferi* | DSM 19554 | | 10±1% | 0±0% |
| *Staphylococcus pettenkoferi* | Vesteras 18:1 | | 51±7% | 0±2% |

**Table S3.** Summary of X-ray data collection, relevant data processing and the model refinement statistics. Values in parentheses are for the highest resolution shell.

| | |
|---|---|
| **Data collection and processing** | |
| Beamline | P13 (DASY, Hamburg) |
| Wavelength (Å) | 0.9116 |
| Resolution range (Å) | 47.79 - 2.99 (3.10 - 2.99) |
| Space group | $P\ 6_5\ 2\ 2$ |
| Unit cell (Å, °) | 136.5, 136.5, 325, 90, 90, 120 |
| Total no. of reflections | 802631 (36807) |
| No. of unique reflections | 36798 (3579) |
| Multiplicity | 21.9 |
| Completeness (%) | 99.84 (99.11) |
| Mean I/σ(I) | 16.6 (1.1) |
| $R_{meas}$ (%) | 21.6 (299.7) |
| $CC_{1/2}$ | 99.9 (43.4) |
| **Refinement** | |
| Reflections in working set | 34966 (3403) |
| Reflections in test set | 1841(179) |
| $R_{work}$ (%) | 22.5 (39.4) |
| $R_{free}$ (%) | 25.9 (42.4) |
| Number of non-H atoms | 4883 |
| macromolecule | 4839 |
| ligands | 5 |
| water | 39 |
| RMS(bonds) (Å) | 0.007 |
| RMS(angles) (°) | 0.96 |
| Ramachandran favored (%) | 94.65 |
| Ramachandran outliers (%) | 0 |
| Rotamer outliers (%) | 0 |
| Clashscore | 7.42 |
| Average B-factor (Å$^2$) | 99.6 |
| macromolecule | 99.8 |
| ligands | 85.9 |
| water | 80.8 |
| Number of TLS groups | 5 |

**Figure S1.** Crystal packing. **(A)** A sphere representation of crystal packing showing the content of the four adjacent unit cells, restricted by lines representing crystal axis labeled with lower cases. Each layer of molecules is arranged around $6_5$ axis (c axis) and is depicted in distinct color. Molecules of one asymmetric unit are labelled A, B, C, D and E. **(B)** A representation of non-crystallographic $5_1$ 2 symmetry. Five molecules of the asymmetric unit A-E are represented by the surface and molecules related by the two-fold crystallographic symmetry A' - E' are shown as a spheres. Molecules AB, CD, EE', D'C' and B'A' create dimers internally related by non-crystallographic two-fold symmetry. **(C)** View along the $5_1$ propagating axis. All dimers are paired by the line to depict non-crystallographic $5_1$ symmetry perpendicular to hexagonal axis. Consecutive dimers are twisted at around 72° angle.

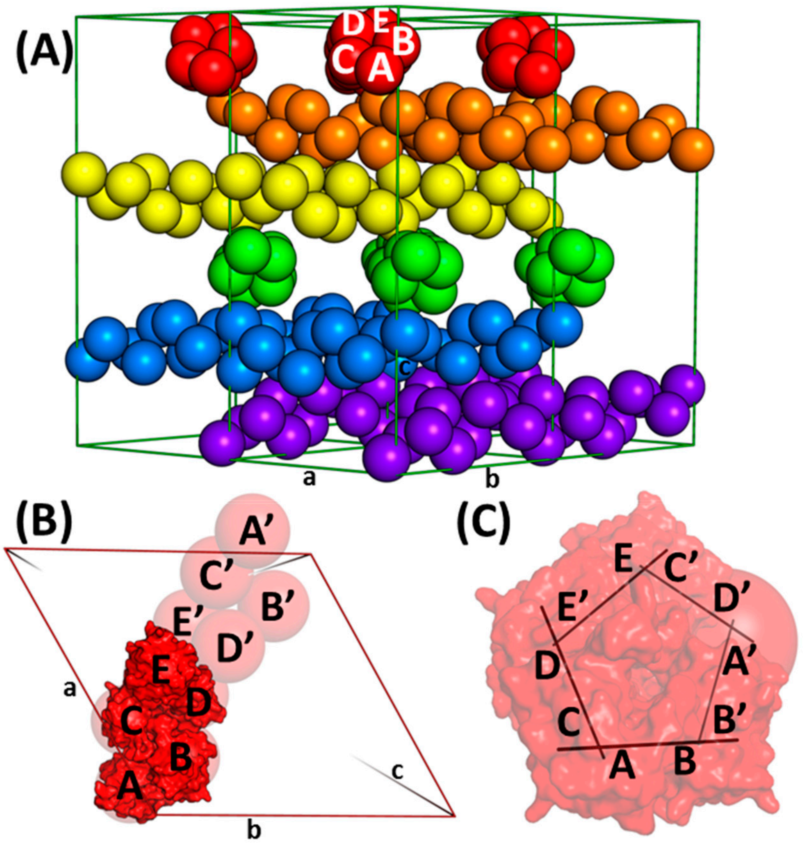


The crystal is composed of layers of molecules arranged around hexagonal axis (**Fig. 2A**). All protein molecules are packed as dimers related by non-crystallographic 2-fold symmetry. Molecules in dimers are propagating along the axis perpendicularly to six fold symmetry. Consecutive dimers rotate by approximately 72° creating a twisted coil (double helix) which implies non-crystallographic $5_1$ screw axis. Together with crystallographic 2-fold axis, it creates $5_1 2$ symmetry **(Fig. 2B**). The crystal contacts connecting separated coils are observed along the crystallographic $6_5$ screw axis and it is the only joint between them **(Fig. 2C)**. Similar helical arrangement of crystalized molecules were reported previously[66].

**Figure S2.** Relevant amino acid residues around an active site, metal ion and water molecule shown as sticks, green sphere and red sphere respectively. The position of atoms are evidenced by the (2mFo-DFc, αc) map in black contoured at 1 σ within 2 A of selected atoms.

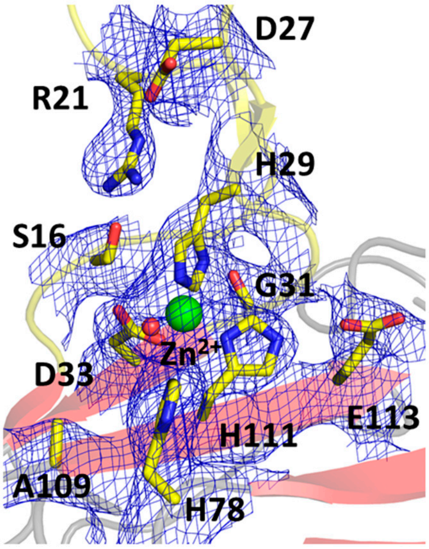

**Figure S3. A** spaghetti representation of superimposed catalytic domains of proteins structurally related to $EnpA_{CD}$. Constant **(A)** and variant **(B)** elements are separated. The core of $EnpA_{CD}$ on (B) is shown as red semi-transparent cartoon; an active site ion as a green sphere. Color code is used as following: $EnpA_{CD}$ - red; M23 from *Vibrio cholerae* (PDB id:2gu1)- orange; NMB0315 from *Neisseria meningitidis* (PDB id: 3slu) - grey; Csd2 from *Helicobacter pylorii* (PDB id:5j1k) - navy blue; LytM (PDB id:1qwy) - magenta; Lss (PDB id:4lxc) - cyan; M23 from *Pseudomonas aeruginosa* (PDB id:2HSI) - lime; Csd3 from *Helicobacter pylori* (PDB id:4rny) - light pink; Csd1 from *Helicobacter pylori* (PDB id:5j1m) - chocolate; channel complex from *Bacillus subtilis* (PDB id:3tuf) light blue, M23 from *Ruminococcus gnavus* (RUMGNA_02482) (PDB id:3nyy) -purple; M23 from *Lactobacillus salivarius* REN (PDB id:5gt1) - black; bacteriophage Φ29 (PDB id:3csq) - green; ZoocinA from *Streptococcus equi* (PDB id:5kvp) - yellow; LasA from *Pseudomonas aeruginosa* (PDB id:3it5) – deep olive; Chemotaxin2 from human leukocyte (PDB id:5B0H) - white; LytU from a *Staphylococcus aureus* (PDB id:KQB) - marine.

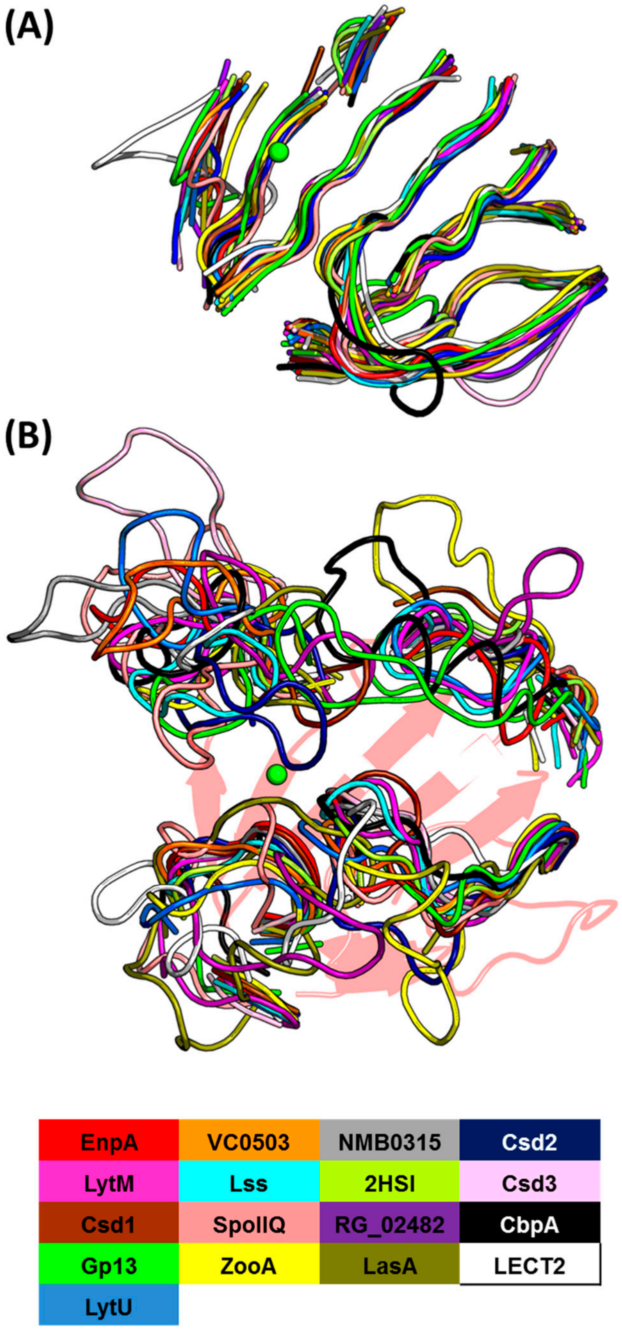

**Figure S4**. Dependence of ionic strength and buffer composition on EnpA$_{CD}$ activity . Showed as results of the lytic test (turbidity reduction assay) carried out on *S. aureus* 8325-4 strain in 50mM glycine buffer pH = 8.0. The enzyme concentration is indicated and each bar represents the mean of three measurements.

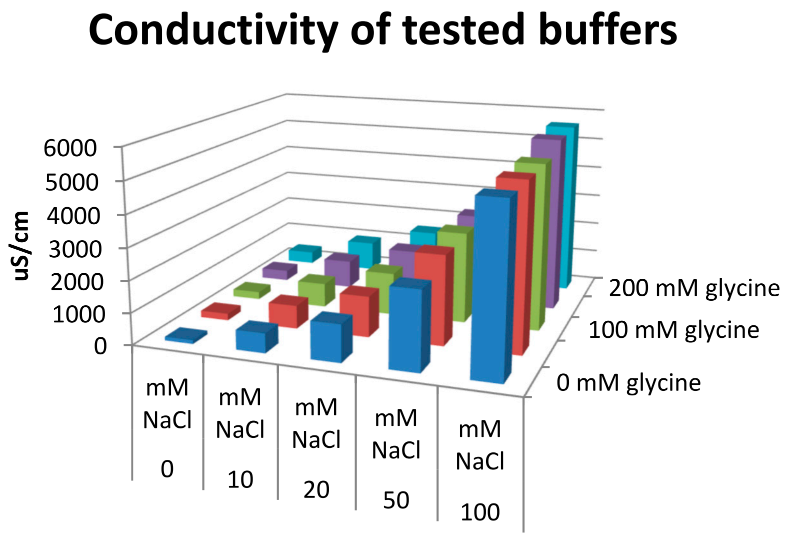


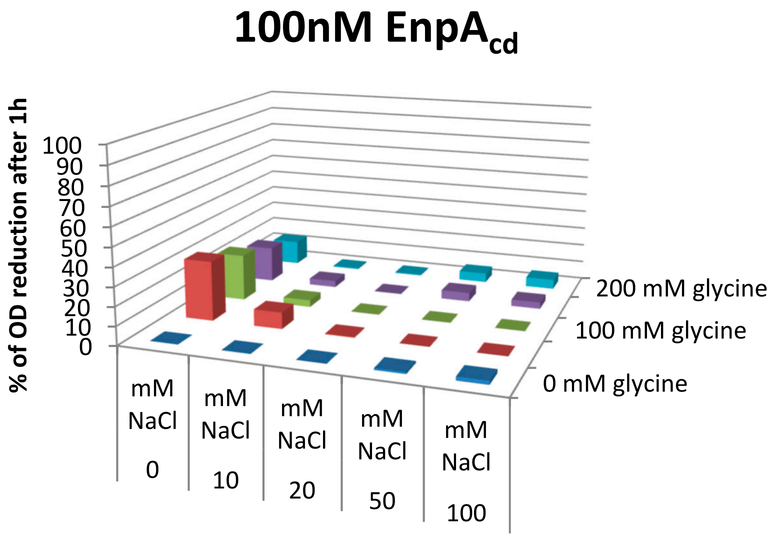

## 500nM $EnpA_{cd}$

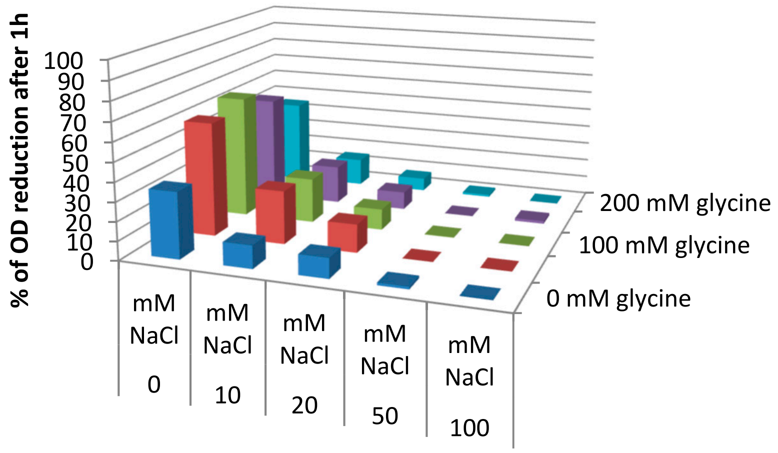


## 1000nM $EnpA_{cd}$

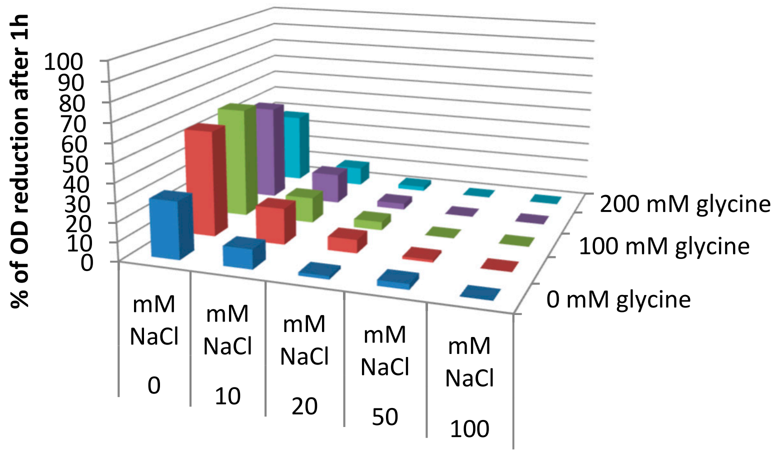


## 2000nM $EnpA_{cd}$

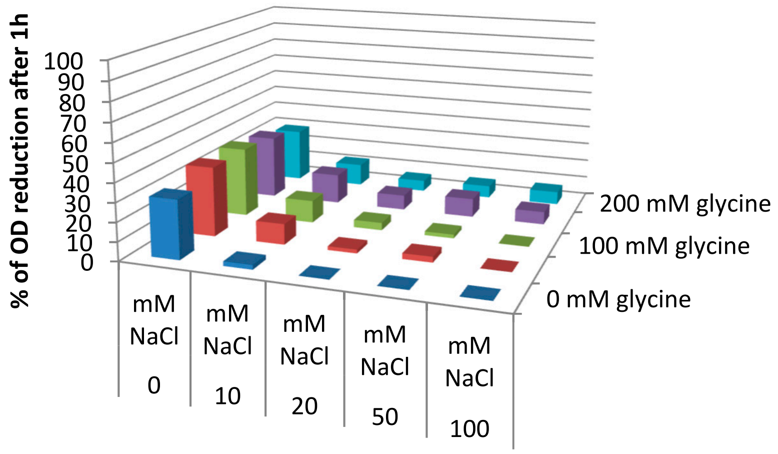

**Figure S5.** The effect of temperature on $EnpA_{CD}$ activity against *S. aureus* 8325-4. Activity was measured using turbidity reduction assay. The measurement was carried out in 1 ml cuvettes, which were incubated at appropriate temperatures. Each bar represents the mean and standard deviation of three independent measurements.

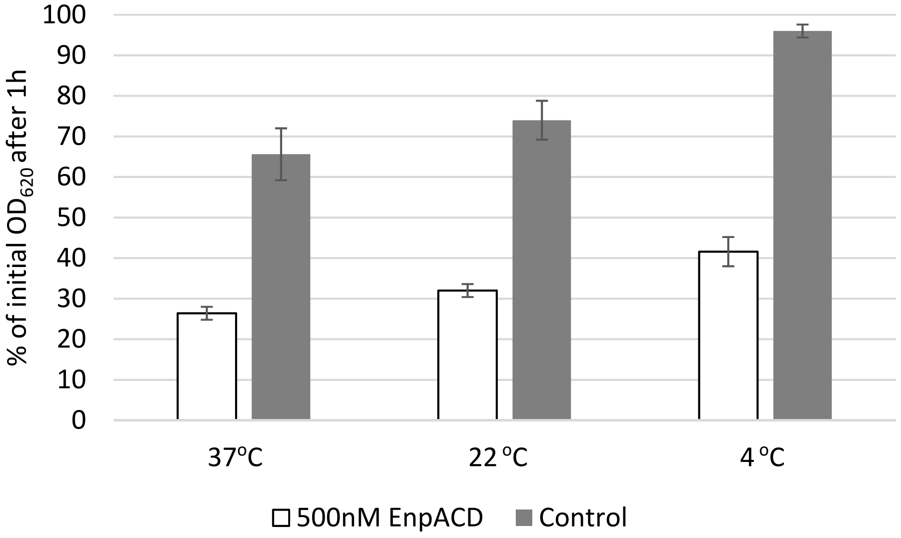

**Figure S6.** The effects of modifications of *S. aureus* cell wall on $EnpA_{CD}$ bacteriolytic activity. Results of the lytic test carried out on *S. aureus 8325-4* in glycine buffer pH = 8.0 for 1 hour. The final concentration of $EnpA_{CD}$ was 500nM, while LytM and Lss - 100nM. Each experiment was repeated three times using an enzyme from the same preparation and different bacterial cultures. Three technical repetitions were prepared each time. **(A)** The effect of the cross-bridge length. **(B)** The effect of serine substitution in cross-bridge. **(C)** The effect of presence of wall teichoic acids. Tests were run on various strains with synthesis of teichoic acids inhibited either by mutation in *tagO* gene or treatment with tunicamycin. The decrease in $OD_{620}$ after one hour was calculated as a percentage of the initial $OD_{620}$ in the control without the enzyme. The decrease in $OD_{620}$, in the control means autolysis, while in the sample the decrease in $OD_{620}$ is due to autolysis and enzyme activity.

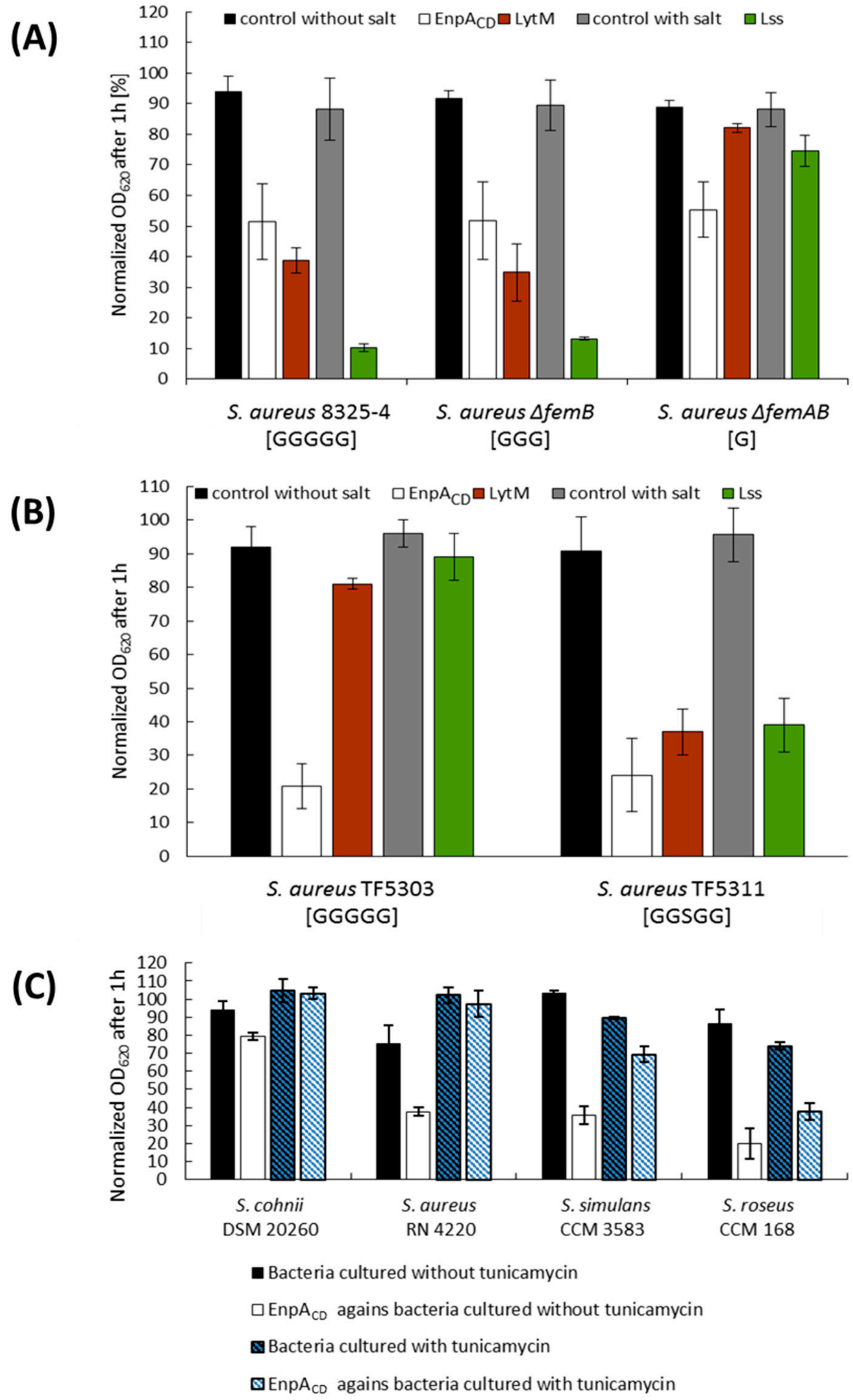

**Figure S7**. Structure of EnpA (pdb ID: 6SMK)  and LysK CHAP domain (pdb ID: 4CSH)

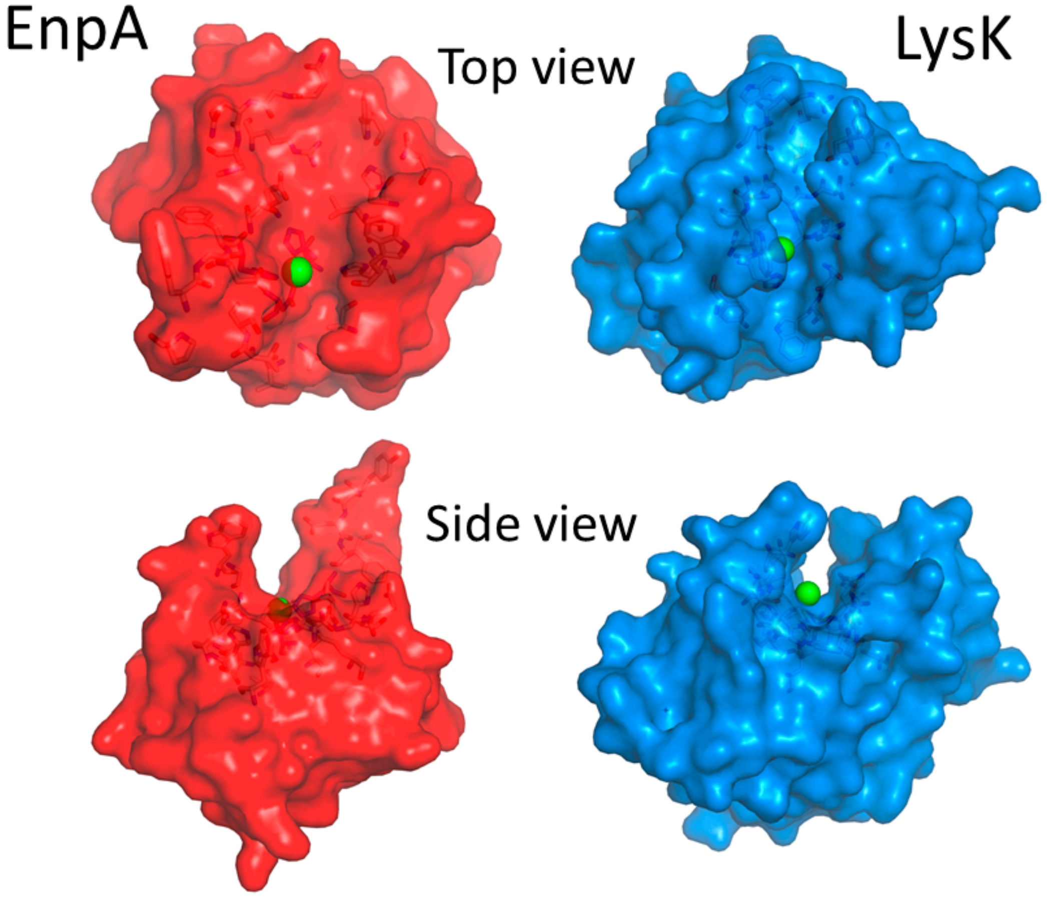


**Figure S8.** Amino acid sequence alignment of M23 enzymes based on structural and sequential constraints done using PROMSAL3D software[67] **.** The loop position is marked by blue rectangles and labeled. Zinc ligands in HxxxD and HxH motis are colored green. Conserved histidine residues taking part in catalysis are colored orange. Arg21 and its structural equivalents are colored red.

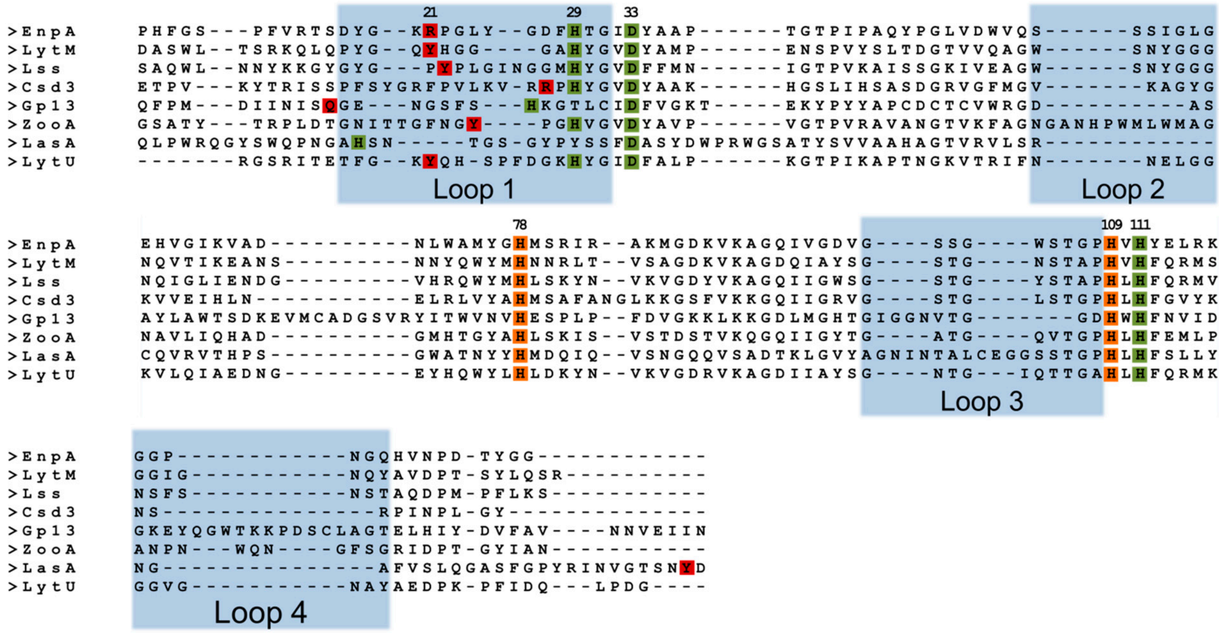
21
29
33
>EnpA PHFGS---PFVRTSDYG--KRPGLY--GDFHTGIDYAAP------TGTPIPAQYPGLVDWVQS------SSIGLG
>LytM DASWL--TSRKQLQPYG--QYHGG----GAHYGVDYAMP------ENSPVYSLTDGTVVQAGW------SNYGGG
>Lss SAQWL--NNYKKGYGYG---PYPLGINGGMHYGVDFFMN------IGTPVKAISSGKIVEAGW------SNYGGG
>Csd3 ETPV---KYTRISSPFSYGRFPVLKV-RRPHYGVDYAAK------HGSLIHSASDGRVGFMGV-------KAGYG
>Gp13 QFPM---DIINISQGE---NGSFS---HKGTLCIDFVGKT-----EKYPYYAPCDCTCVWRGD----------AS
>ZooA GSATY---TRPLDTGNITTGFNGY----PGHVGVDYAVP------VGTPVRAVANGTVKFAGNGANHPWMLWMAG
>LasA QLPWRQGYSWQPNGAHSN-----TGS-GYPYSSFDASYDWPRWGSATYSVVAAHAGTVRVLSR------------
>LytU -------RGSRITETFG--KYQH-SPFDGKHYGIDFALP------KGTPIKAPTNGKVTRIFN-------NELGG
Loop 1
Loop 2
78
109
111
>EnpA EHVGIKVAD----------NLWAMYGHMSRIR--AKMGDKVKAGQIVGDVG----SSG----WSTGPHVHYELRK
>LytM NQVTIKEANS---------NNYQWYMHNNRLT--VSAGDKVKAGDQIAYSG----STG----NSTAPHVHFQRMS
>Lss NQIGLIENDG---------VHRQWYMHLSKYN--VKVGDYVKAGQIIGWSG----STG----YSTAPHLHFQRMV
>Csd3 KVVEIHLN-----------ELRLVYAHMSAFANGLKKGSFVKKGQIIGRVG----STG----LSTGPHLHFGVYK
>Gp13 AYLAWTSDKEVMCADGSVRYITWVNVHESPLP--FDVGKKLKKGDLMGHTGIGGNVTG-------GDHWHFNVID
>ZooA NAVLIQHAD----------GMHTGYAHLSKIS--VSTDSTVKQGQIIGYTG----ATG----QVTGPHLHFEMLP
>LasA CQVRVTHPS----------GWATNYYHMDQIQ--VSNGQQVSADTKLGVYAGNINTALCEGGSSTGPHLHFSLLY
>LytU KVLQIAEDNG---------EYHQWYLHLDKYN--VKVGDRVKAGDIIAYSG----NTG---IQTTGAHLHFQRMK
Loop 3
>EnpA GGP------------NGQHVNPD-TYGG------------
>LytM GGIG-----------NQYAVDPT-SYLQSR----------
>Lss NSFS-----------NSTAQDPM-PFLKS-----------
>Csd3 NS---------------RPINPL-GY--------------
>Gp13 GKEYQGWTKKPDSCLAGTELHIY-DVFAV----NNVEIIN
>ZooA ANPN---WQN----GFSGRIDPT-GYIAN-----------
>LasA NG---------------AFVSLQGASFGPYRINVGTSNYD
>LytU GGVG-----------NAYAEDPK-PFIDQ---LPDG----
Loop 4

**Figure S9.** To evaluate the role of arginine R21 in EnpA$_{CD}$ activity we have generated a protein with substitution R21Y which showed no activity against tested bacterial strains (A). FTIR confirmed that introduced substitution does not have a negative effect on protein folding as the overall structure is preserved (B).

**(A)** Activity of EnpA$_{CD}$R21Y variant.

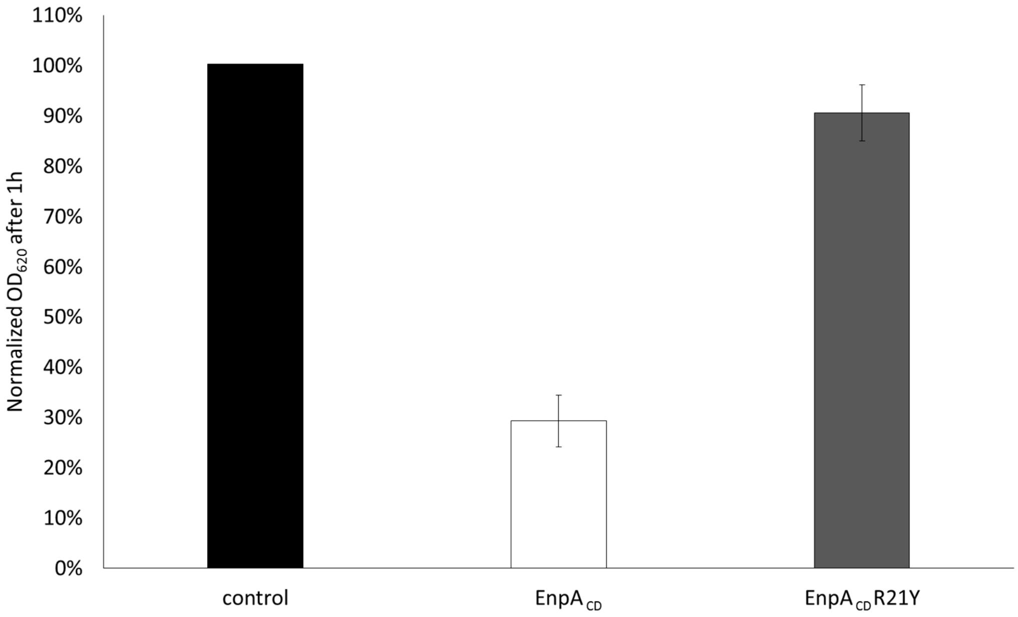


**(B)** Results of FTIR measurement. The graph shows the normalized absorbance for wild-type EnpA$_{CD}$ and the R21Y variant. The table shows the percentage of secondary structures in a particular protein.

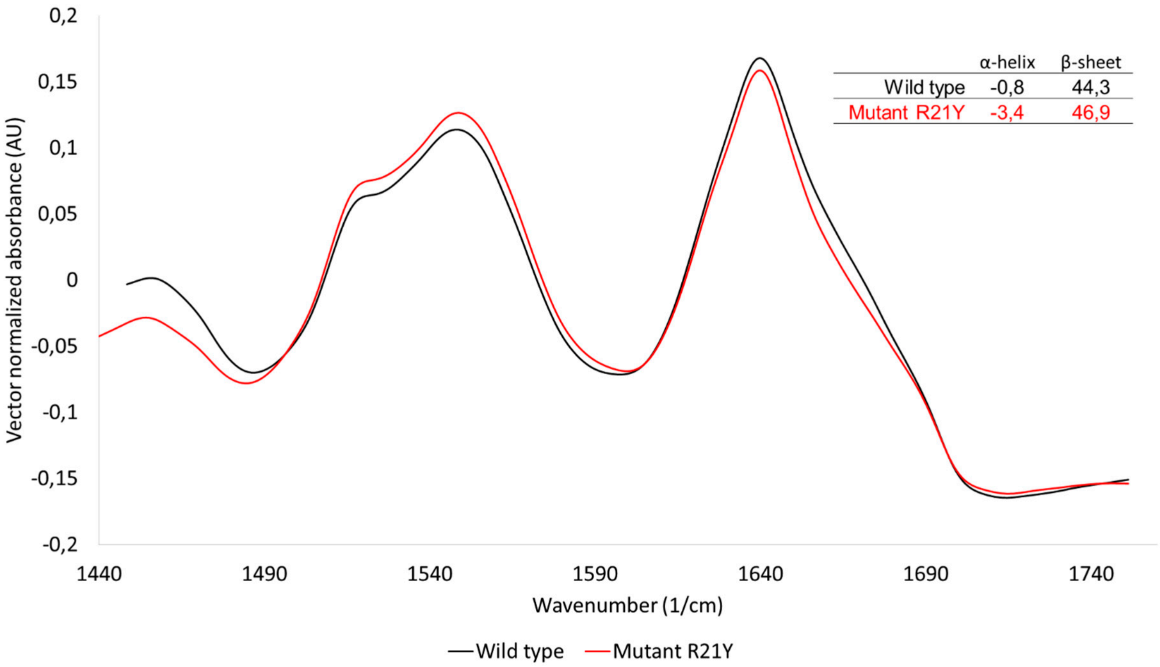

Supplement: Supplementary file 1 [file ijms-22-07136-s001.zip › ijms-1247363-supplementary.pdf]
